# Optimal prediction of peritoneal dialysis to hemodialysis transition: a penalized fine-gray model

**DOI:** 10.3389/fmed.2026.1843381

**Published:** 2026-10-21

**Authors:** Jingfang Wan, Qingyu Wei, Dong Wu, Shihui Hou, Kaizhen Yv, Rui Tang, Jian He, Xuelian Chen, Yang Li, Yulin Hu, Jie Yang, Tingting Li, Kehong Chen

**Affiliations:** 1 Department of Nephrology, Daping Hospital, Army Medical University, Chongqing, China; 2 School of Mathematics and Statistic, Southwest University, Chongqing, China; 3 Department of Nephrology, Kaizhou District People’s Hospital, Chongqing, China; 4 Department of Nephrology, Yongchuan Hospital of Chongqing Medical University, Chongqing, China; 5 State Key Laboratory of Trauma, Burns and Combined Injury, Wound Trauma Medical Center, Army Medical University, Chongqing, China

**Keywords:** competing risks, fine-gray model, hemodialysis, nomogram, peritoneal dialysis

## Abstract

**Background:**

Traditional Cox proportional hazards models overestimate the risk of progression from peritoneal dialysis (PD) to hemodialysis (HD). This study aims to screen high-risk conversion populations using competing risk models.

**Methods:**

In this multicenter retrospective cohort study, we enrolled patients aged ≥16 years who were undergoing regular PD for >1 month; transition from PD to HD was the target event. When death was recognized as a competing event, we employed cause-specific proportional hazards and Fine-Gray models for multivariate analysis. A conventional LASSO-Cox model was also established for comparative purposes. Model performance was evaluated using time-dependent area under the curve (AUC) and C-index curves. The best model was identified to construct the cumulative incidence nomogram for PD to HD at the 3-, 6-, and 9-year time points.

**Results:**

We enrolled 700 valid samples that were randomly allocated at a ratio of 7:3 to the training (*n* = 491) and test sets (*n* = 209). The penalized Fine-Gray model was identified as the optimal model (AUC: 0.716, C-index: 0.662). Body mass index, history of peritonitis hospitalization, and nutrition-related parameters were identified as optimal predictors for transitioning from PD to HD. AUC values for the penalized Fine-Gray model at 3, 6, and 9 years were 0.675, 0.715, and 0.714, respectively. We constructed a nomogram for 3, 6, and 9 years based on the penalized Fine-Gray model, which demonstrated significant clinical utility.

**Conclusion:**

The penalized Fine-Gray model serves as the optimal choice for predicting the transition from PD to HD, and the nomogram constructed based on this model exhibits favorable clinical applicability.

## Introduction

Peritoneal dialysis (PD) is a common renal replacement therapy in patients with chronic renal failure. Over 900,000 individuals in Asia currently undergo PD, and this number is projected to triple by 2030 (1). PD offers numerous advantages, including a delayed decline in residual renal function, enhanced social participation, and reduced healthcare expenditures (2, 3).

Our previous study revealed a relatively high incidence of adverse events associated with PD (4). Studies show that 20–35.7% of patients with PD transition to hemodialysis (HD) due to peritonitis, PD catheter dysfunction, adequacy issues, or other reasons (5–7). Compared to PD treatment costs, HD treatment medical expenditures have shown a significant increase (8, 9). Moreover, after transitioning from PD to HD, the crude mortality rate in the first month reached 30%–60% patient-years, with a mortality rate as high as 25% after 24 months (6, 9). Thus, early identification of high-risk patients for conversion can enable precise stratified management and implementation of preventive intervention strategies, potentially delaying or preventing disease progression, thereby reducing conversion risk and healthcare expenditures.

Traditional Cox proportional hazards models focus exclusively on the transition from PD to HD and treat death as a censored event, which consequently leads to an overestimation of the patients’ HD transition risk (10–12). When death is recognized as a competing event, competing risk models become applicable, and have been applied in multiple clinical scenarios. Compared to traditional survival analyses, this model has been shown to yield lower mortality rates in patients with rectal cancer. The C-statistic of the model reached 0.821 for the training set and 0.829 for the validation set (13). A competing risks model for predicting small-for-gestational-age (SGA) fetuses in the second trimester treated deliveries at different gestational weeks (<37, <40, and <42 weeks) as competing events. At a fixed false-positive rate (10%), it demonstrated high detection rates for SGA fetuses across gestational ages (88.0% for SGA fetus <3rd percentile delivered at <37 weeks) (14). Furthermore, the competing risk model is widely applied to measles, preeclampsia, cardiovascular diseases in the elderly, and renal transplant failure, providing a critical reference for guiding the clinical screening of high-risk populations and decision-making (15–17). In this study, we retrospectively collected demographic characteristics, laboratory data, and clinical data of patients undergoing PD at multiple centers. Using two competing risk models, the cause-specific proportional hazards (CSH) model and the Fine-Gray model, we explored the early risk factors for the transition from PD to HD, constructed an optimal predictive model, identified high-risk populations early, and implemented proactive interventions to improve patient outcomes.

## Methods

### Data sources and study population

Our data were sourced from three dialysis centers: Daping Hospital, Kaizhou District People's Hospital, and Yongchuan Hospital of Chongqing Medical University.

Clinical data were retrieved from the hospital electronic medical record system for patients who initiated PD for the first time between August 2007 and September 2025, yielding 824 records. The inclusion criteria were as follows: (1) confirmed diagnosis of chronic renal failure and receipt of regular PD therapy for >1 month; (2) age ≥16 years; and (3) no sex restrictions. The exclusion criteria were as follows: (1) patients with acute kidney injury, those who underwent emergency PD, or those with subsequent renal function recovery; (2) patients who underwent kidney transplantation during the follow-up period; and (3) patients lost to follow-up.

In the primary analysis, we excluded patients who recovered renal function (*n* = 1, 0.12%) and those who underwent kidney transplantation (*n* = 36, 4.4%), totaling 37 patients (4.5% of the cohort). This decision was based primarily on statistical feasibility: the number of events in each of these two categories was small, particularly for renal function recovery (*n* = 1). Competing-risk models (e.g., the Fine-Gray model) require a sufficient number of events within each competing category to ensure the stability of subdistribution hazard ratio estimates and to permit verification of the proportional hazards assumption; incorporating multiple candidate predictors into categories with limited events could yield highly unstable estimates with unacceptably wide confidence intervals, thereby undermining rather than strengthening the reliability of the model. Patients lost to follow-up (*n* = 44, 5.3%), whose true outcomes were unknown, were treated as censored observations and were not treated as competing events.

Based on the above criteria, 737 patients with PD were enrolled in this study. Outliers were identified using the mean ± 5 standard deviations method, and 37 samples were excluded, yielding 700 valid cases. These valid samples were then randomly allocated to a training set (*n* = 491) or test set (*n* = 209) at a ratio of 7:3. The detailed patient screening flowchart is presented in Figure 1.

**Figure 1:**
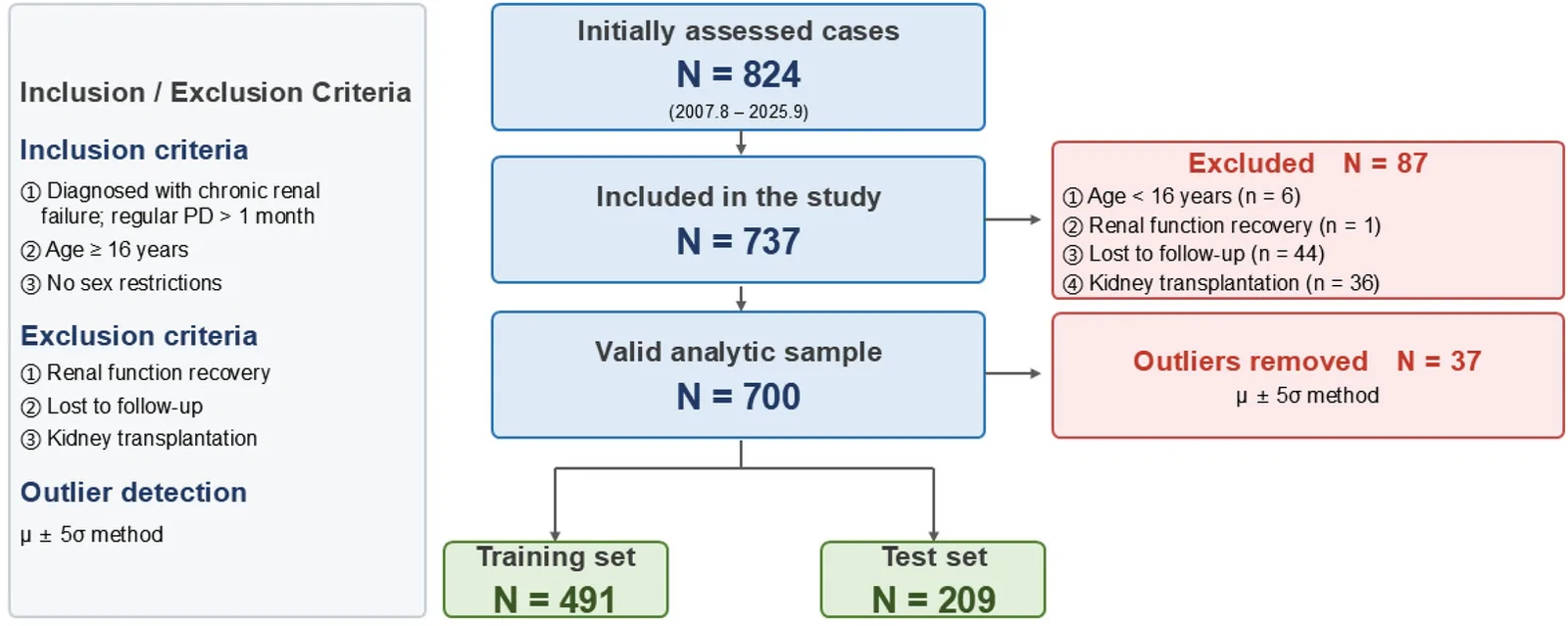
Flow chart of patient screening and enrollment.

According to the 2026 The International Society for Peritoneal Dialysis (ISPD) Position Statement (18), HD transfer is defined as a transition from PD to HD lasting more than 90 days without return to PD. If a patient experiences death during the 90-day observation period, the event will not be classified as HD transfer; instead, they were analyzed as death events.

### Characteristics inclusion

Based on the principles of physiological relevance and inter-parameter interactions, all clinical variables were further summarized into nine composite domains. Detailed results are presented in Table 1.

**Table 1:** Critic weight.

Comprehensive characteristics	Clinical characteristics	Weight
Anemia-related parameters	Hemoglobin	0.34
	Ferritin	0.34
	Transferrin saturation	0.32
Calcium-phosphorus metabolism	Calcium-phosphorus product	0.25
	Intact parathormone	0.4
	Alkaline phosphatase	0.35
Nutrition-related parameters	Serum albumin	0.49
	Prealbumin	0.51
Electrolyte-related parameters	Serum sodium	0.2
	Serum potassium	0.27
	Serum chloremia	0.23
	Carbon dioxide combining power	0.3
Renal function-related parameters	Serum creatinine	0.21
	estimated glomerular filtration rate	0.24
	Serum urea nitrogen	0.24
	Uric acid	0.31
Lipid-related parameters	Total cholesterol	0.22
	Triglyceride	0.3
	Low-density lipoprotein	0.24
	High-density lipoprotein	0.24
Glycemic control indicators	Fasting glycemic control	0.43
	Glycosylated hemoglobin	0.57
Cardiac function-related parameters	Troponin	0.35
	Creatine kinase	0.34
	Myoglobin	0.31

Missing values were concentrated in laboratory variables and followed a multivariate non-monotone missing pattern (19). Variables with a missing data rate exceeding 60% and categorical variables with an extremely sparse distribution (i.e., fewer than four cases per category) were excluded from subsequent analyses. For the remaining missing data, multiple imputation strategies were applied based on the extent of missingness and variable relationships: (1) mean imputation was adopted for variables with a missing rate below 10%; (2) K-nearest neighbor (KNN) imputation was employed for physiologically correlated and interacting variables; and (3) multiple linear regression imputation was used when a set of correlated variables exhibited concurrent high missing rates.

The baseline characteristics of the study cohort are summarized in Table 2, where continuous variables are expressed as mean ± standard deviation and categorical variables as frequency (percentage). The missing rate of each variable and the corresponding imputation methods are presented in Supplementary Table S1.

**Table 2:** Baseline characteristics of study population.

Characteristics	Overall (700)	HD Transition (320)	Death (178)	Keep PD (202)
Gender (Male)	402 (57%)	204 (63.7%)	84 (47.2%)	98 (48.5%)
Age (years)	48.83 ± 15.1	47.00 ± 14.20	58.24 ± 15.14	43.43 ± 12.59
Ethnicity (Han)	664 (95%)	303 (94.7%)	171 (96.1%)	190 (94.1%)
Marital status (No Spouse)	44 (6%)	21 (6.6%)	6 (3.4%)	17 (8.4%)
Education level				
<6 years	166 (24%)	78 (24.4%)	67 (37.6%)	21 (10.4%)
6–9 years	257 (37%)	125 (39.1%)	53 (29.8%)	79 (39.1%)
9–12 years	132 (19%)	58 (18.1%)	25 (14.0%)	49 (24.3%)
>12 years	145 (21%)	59 (18.4%)	33 (18.5%)	53 (26.2%)
Smoking history (No)	522 (75%)	237 (74.1%)	132 (74.2%)	153 (75.7%)
Drinking history (No)	587 (84%)	267 (83.4%)	150 (84.3%)	170 (84.2%)
Blood pressure (Normal)	239 (34%)	105 (32.8%)	65 (36.5%)	69 (34.2%)
Heart rate (Normal)	586 (84%)	277 (86.6%)	156 (87.6%)	153 (75.7%)
24 H urine output				
Extremely low	51 (7%)	23 (7.2%)	20 (11.2%)	8 (4.0%)
Low	136 (19%)	64 (20.0%)	37 (20.8%)	35 (17.3%)
Normal	473 (68%)	211 (65.9%)	116 (65.2%)	146 (72.3%)
High	40 (6%)	22 (6.9%)	5 (2.8%)	13 (6.4%)
BMI (kg/m^2^)	23 ± 3.6	23.21 ± 3.58	22.99 ± 3.66	22.69 ± 3.59
Primary renal disease				
Chronic glomerulonephritis	402 (57%)	184 (57.5%)	82 (46.1%)	136 (67.3%)
Diabetic kidney disease	101 (14%)	46 (14.4%)	44 (24.7%)	11 (5.4%)
Others	65 (9%)	29 (9.1%)	22 (12.4%)	14 (6.9%)
Unknown	132 (19%)	61 (19.1%)	30 (16.9%)	41 (20.3%)
CCD (No)	621 (89%)	284 (88.8%)	144 (80.9%)	193 (95.5%)
Hypertension (No)	314 (45%)	142 (44.4%)	72 (40.4%)	100 (49.5%)
Diabetes mellitus (No)	581 (83%)	271 (84.7%)	125 (70.2%)	185 (91.6%)
History of urological surgery (No)	689 (98%)	314 (98.1%)	177 (99.4%)	198 (98.0%)
History of kidney transplantation (No)	694 (99%)	316 (98.8%)	176 (98.9%)	202 (100.0%)
History of haemodialysis (No)	672 (96%)	313 (97.8%)	162 (91.0%)	197 (97.5%)
Peritoneal catheter surgery plan				
Percutaneous catheter placement Surgery	56 (8%)	22 (6.9%)	10 (5.6%)	24 (11.9%)
Open surgery	644 (92%)	298 (93.1%)	168 (94.4%)	178 (88.1%)
Antidiabetic medications (No)	601 (86%)	275 (85.9%)	135 (75.8%)	191 (94.6%)
LLM (No)	612 (87%)	277 (86.6%)	159 (89.3%)	176 (87.1%)
UALM (No)	608 (87%)	291 (90.9%)	163 (91.6%)	154 (76.2%)
CPMM (No)	232 (33%)	105 (32.8%)	61 (29%)	57 (28.2%)
Anemia medications (No)	165 (24%)	76 (23.8%)	51 (24%)	40 (19.8%)
Antihypertensive medications (No)	104 (15%)	38 (11.9%)	35 (17%)	35 (17.3%)
History of hospitalization for peritonitis (No)	462 (66%)	161 (50.3%)	124 (59%)	167 (82.7%)
History of hospitalization for cardiovascular disease (No)	601 (86%)	275 (85.9%)	177 (85%)	187 (92.6%)
Anemia-related parameters	0.28 ± 0.17	0.29 ± 0.10	0.28 ± 0.13	0.28 ± 0.10
Calcium-phosphorus metabolism	0.8 ± 0.12	0.8 ± 0.11	0.79 ± 0.13	0.79 ± 0.11
Nutrition-related parameters	0.48 ± 0.12	0.48 ± 0.12	0.45 ± 0.12	0.52 ± 0.11
Electrolyte-related parameters	0.89 ± 0.1	0.88 ± 0.10	0.88 ± 0.11	0.89 ± 0.09
Renal function-related parameters	0.6 ± 0.1	0.6 ± 0.1	0.63 ± 0.09	0.58 ± 0.10
Lipid-related parameters	0.57 ± 0.09	0.56 ± 0.1	0.58 ± 0.09	0.57 ± 0.09
Glycemic control indicators	0.71 ± 0.09	0.7 ± 0.09	0.68 ± 0.11	0.73 ± 0.07
Cardiac function-related parameters	0.88 ± 0.08	0.91 ± 0.15	0.92 ± 0.11	0.93 ± 0.13
Infection-related parameters	0.92 ± 0.13	0.88 ± 0.08	0.87 ± 0.08	0.88 ± 0.08

PD, peritoneal dialysis; HD, hemodialysis; BMI, body mass index; CCD, cardiovascular and cerebrovascular diseases; LLM, lipid-lowering medications; UALM, uric acid-lowering medications; CPMM, calcium-phosphorus metabolism medications. Blood pressure: Normal blood pressure is defined as systolic pressure <140 and diastolic pressure <90; otherwise, it is considered hypertension. Heart rate: <100 is normal, ≥100 is rapid. 24 H urine output: <400 is extremely low, 400–1,000 is low, 1,000–2,000 is normal, >2,000 is high. CCD include congestive heart failure, coronary heart disease, arrhythmia, peripheral artery disease, and myocardial infarction. Antihypertensive medications include ARB (Angiotensin receptor blockers), ACEI (Angiotensin-converting enzyme inhibitors), CCB (Calcium channel blockers), beta-blockers, alpha-blockers, alpha/beta-blockers, and diuretics. CPMM include calcium-phosphate binders, active vitamin D, non-calcium-phosphate binders and calcimimetics. Anemia medications include iron supplements and EPO (Erythropoietin).

### Development and evaluation of models

Two competing risk models and a conventional proportional hazards model were established: the cause-specific proportional hazards (CSH) model (20), Fine-Gray model (21) and LASSO-Cox model. All models were constructed using R software (version 4.5.1), with the employed packages including riskRegression, fastcmprsk, CoxBoost, pec, and QHScrnomo. All statistical tests were two-tailed, and statistical significance was defined as *p* < 0.05.

The CSH was combined with least absolute shrinkage and selection operator (LASSO) regression for feature selection. Tenfold cross-validation was adopted, and the concordance index (C-index) was used as the optimization criterion. For the Fine-Gray model, two approaches were integrated for feature selection to establish the final model: penalized regression (LASSO, SCAD, and MCP) (22, 23) and machine learning-based boosting (24). For the penalized regression step, the Akaike Information Criterion (AIC) of the model was set as the optimization target. In the boosting method, features that were statistically significant in the univariate analysis were designated as forced features, whereas the remaining features were treated as optional features. For the LASSO-Cox model, variables were first screened via AIC-optimized LASSO penalized regression, with death treated as conventional censoring, followed by stepwise regression to fit the final Cox model.

Time-dependent area under the curve (AUC) curves (25) and C-index curves were used to evaluate the predictive performance of the models and to identify the optimal model setup. The detailed process of model development and evaluation is illustrated in Figure 2.

**Figure 2:**
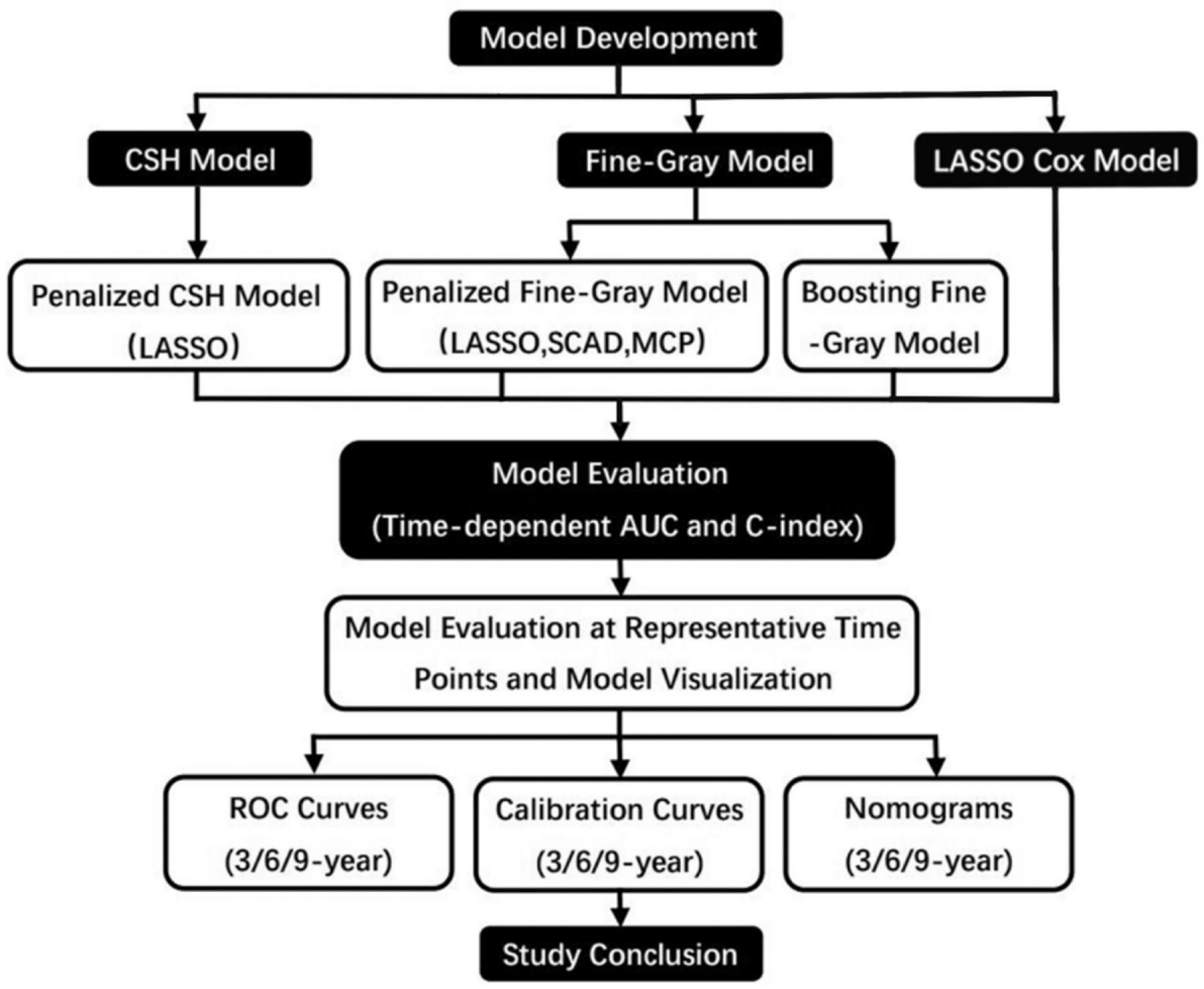
Model development and evaluation flowchart. AUC, area under the receiver operating characteristic curve; C-index, concordance index; CSH, cause-specific hazard; LASSO, least absolute shrinkage and selection operator; MCP, minimax concave penalty; ROC, receiver operating characteristic; SCAD, smoothly clipped absolute deviation.

We plotted the nomogram based on the optimal model. Patients were stratified into low-risk, middle-risk, and high-risk subgroups according to the tertiles of the nomogram total scores derived from the training cohort: low risk, middle risk, and high risk. In the test cohort, cumulative incidence function (CIF) curves of the target and competing events were plotted for each risk subgroup. Gray's test was applied to compare between-group differences in cumulative incidence. The risk stratification performance of the nomogram was evaluated by examining the ordering of CIF curves across subgroups.

## Results

The conventional process was initially adopted during the feature selection phase of the competing risk models. For all potential prognostic features, a univariate analysis was performed to preliminarily screen for those with statistically significant results, as presented in Table 3.

**Table 3:** Univariate test results with significant characteristics .

CSH model		Fine-Gray model	
Characteristics	*P* Value	Characteristics	*P* Value
History of hospitalization for peritonitis	<0.001	History of hospitalization for peritonitis	<0.001
Antidiabetic medications	<0.001	Gender	0.008
BMI	0.001	BMI	0.007
Gender	0.003	History of hemodialysis	0.011
Nutrition-related parameters	<0.001	LLM	0.037
–	–	Lipid-related parameters	0.041

CSH, Cause-specific proportional hazards; BMI, body mass index; LLM, Lipid-lowering medications.

Notably, a relatively small number of significant features were identified using univariate analysis alone. Confining the model construction to these screening results may lead to the oversight of important features with potential clinical or statistical associations. Instead of excluding all insignificant variables, this study incorporated other potentially relevant features via feature selection methods while retaining the significant features derived from the univariate analysis. These features were then used to construct multivariate competing risk models, which reduced the potential information loss caused by the removal of features with insufficient statistical significance, and facilitated the development of a more comprehensive and robust competing risk prediction model.

### CSH model

Combined with the LASSO regression for feature selection, the CSH model was constructed, and the results are presented in Table 4. Features including sex, educational level, body mass index (BMI), history of hospitalization due to peritonitis, and nutrition-related parameters were significantly associated with the risk of transitioning (HD) transition (all *P*-values < 0.05). Among these, an educational level of >6 years and good nutritional status acted as protective factors. In contrast, male sex, higher BMI, and a history of hospitalization for peritonitis were associated with an increased instantaneous risk of HD transition and were thus classified as risk factors.

**Table 4:** CSH model results.

Characteristics	Target event: HD transition		Target event: Death	
	Coef	HR (95%CI)	Coef	HR (95%CI)
Gender (Male)	0.37^a^	1.44 (1.07–1.95)	0.28	1.33 (0.89–1.99)
Education level				
6–9 years	−0.51^a^	0.6 (0.42–0.85)	−1.12^a^	0.33 (0.21–0.52)
9–12 years	−0.75^a^	0.47 (0.31–0.72)	−1.42^a^	0.24 (0.13–0.43)
>12 years	−0.44^a^	0.64 (0.43–0.97)	−0.92^a^	0.4 (0.23–0.69)
Heart rate	−0.2	0.82 (0.55–1.22)	−0.37	0.69 (0.4–1.2)
24 H urine output				
Low	0.06	1.06 (0.55–2.04)	−0.23	0.8 (0.39–1.61)
Normal	−0.07	0.93 (0.51–1.72)	−0.31	0.73 (0.38–1.41)
High	0.17	1.19 (0.54–2.59)	−1.55^a^	0.21 (0.06–0.79)
BMI	0.04 ^a^	1.04 (1–1.09)	−0.01	0.99 (0.94–1.05)
Antihypertensive Medications	0.28	1.33 (0.88–2.01)	0.15	1.16 (0.68–2)
Antidiabetic medications	0.38	1.46 (0.99–2.16)	1.16^a^	3.19 (1.99–5.13)
LLM	0.09	1.09 (0.73–1.63)	−0.58	0.56 (0.28–1.12)
History of hospitalization for cardiovascular disease	−0.28	0.75 (0.5–1.13)	0.37	1.45 (0.91–2.32)
History of hospitalization for peritonitis	0.6^a^	1.81 (1.38–2.38)	−0.56^a^	0.57 (0.38–0.86)
Calcium-phosphorus Metabolism	0.29	1.34 (0.4–4.51)	0.42	1.52 (0.32–7.16)
Nutrition-related parameters	−2.1^a^	0.12 (0.04–0.4)	−3.45^a^	0.03 (0.01–0.17)
Lipid-related parameters	−0.99	0.37 (0.08–1.65)	1.34	3.84 (0.51–28.77)

HR, Hazard ratio; CI, Confidence interval; PD, peritoneal dialysis; HD, hemodialysis; BMI, body mass index; LLM, Lipid-lowering medications.

a After the coefficient indicates that the *p*-value of the feature is <0.05. For the categorical characteristics, the first category is the reference level.

### Fine-gray model

The results of feature selection for the Fine-Gray model are presented in Table 5. Features selected via different penalized regression methods (LASSO, SCAD, and MCP) showed high similarity; thus, the union of these features was used to construct a Fine-Gray model. Tables 6, 7 present the results of the Fine-Gray models built using two distinct feature selection approaches (penalized regression and machine learning-based boosting). Notably, some features exhibit a similar direction (sign) and magnitude of their effects in both the penalized and boosting Fine-Gray models. Specifically, age, BMI, history of urological surgery, history of HD, history of hospitalization for peritonitis, and nutrition-related parameters were found to have statistically significant associations with the risk of the actual occurrence of HD transition (all *P*-values < 0.05). Age, history of HD, and good nutritional status were protective factors. In contrast, a higher BMI, history of urological surgery, and history of hospitalization for peritonitis were identified as risk factors.

**Table 5:** Characteristics selection.

Number	LASSO	SCAD	MCP	Boosting
1	Gender	Gender	Gender	Gender
2	Age	Age	Age	Age
3	Education level	Education level	Education level	Education level
4	Heart rate	Heart rate	Heart rate	Blood pressure
5	24 H urine output	24 H urine output	24 H urine output	Heart rate
6	BMI	BMI	BMI	24 H urine output
7	History of urological surgery	History of urological surgery	History of urological surgery	BMI
8	History of hemodialysis	History of hemodialysis	History of hemodialysis	Primary renal disease
9	LLM	Peritoneal Catheter Surgery Plan	Peritoneal Catheter Surgery Plan	Hypertension
10	Antihypertensive medications	LLM	LLM	Diabetes mellitus
11	History of hospitalization for peritonitis	Anemia medications	History of hospitalization for peritonitis	History of urological surgery
12	History of hospitalization for cardiovascular disease	History of hospitalization for peritonitis	History of hospitalization for cardiovascular disease	History of kidney transplantation
13	Nutrition-related parameters	History of hospitalization for cardiovascular disease	Nutrition-related parameters	History of hemodialysis
14	Lipid-related parameters	Nutrition-related parameters	Lipid-related parameters	Peritoneal Catheter Surgery Plan
15	–	Lipid-related parameters	–	Antidiabetic medications
16	–	–	–	LLM
17	–	–	–	Anemia medications
18	–	–	–	Antihypertensive medications
19	–	–	–	History of hospitalization for peritonitis
20	–	–	–	History of hospitalization for cardiovascular disease
21	–	–	–	Nutrition-related parameters
22	-	-	-	Lipid-related parameters

BMI, body mass index; LLM, Lipid-lowering medications.

**Table 6:** Penalized fine-gray model results.

Characteristics	Coef	SHR (95%CI)	*P* Value
Gender	0.27	1.31 (0.97–1.78)	0.075
Age	−0.02	0.98 (0.97–1)	0.003*
Education level			
6–9 years	−0.27	0.77 (0.54–1.1)	0.14
9–12 years	−0.58	0.56 (0.36–0.87)	0.01*
>12 years	−0.41	0.67 (0.43–1.04)	0.071
Heart rate	−0.33	0.72 (0.48–1.08)	0.11
24 H urine output			
Low	−0.2	0.82 (0.45–1.5)	0.52
Normal	−0.27	0.77 (0.45–1.3)	0.32
High	0.22	1.25 (0.64–2.44)	0.51
BMI	0.05	1.05 (1–1.09)	0.037*
History of urological surgery	0.85	2.33 (1.42–3.85)	0.001*
History of hemodialysis	−1.2	0.3 ((0.09–0.98)	0.046*
Peritoneal dialysis catheter surgery plan (Open surgery)	−0.41	0.67 (0.38–1.16)	0.15
LLM	0.24	1.27 (0.85–1.9)	0.24
Antihypertensive Medications	0.23	1.25 (0.83–1.9)	0.28
Anemia-related parameters	0.2	1.22 (0.89–1.68)	0.21
History of hospitalization for peritonitis	0.77	2.15 (1.64–2.84)	0.000*
History of hospitalization for cardiovascular disease	−0.19	0.82 (0.57–1.2)	0.31
Nutrition-related parameters	−1.31	0.27 (0.08–0.9)	0.033*
Lipid-related parameters	−1.14	0.32 (0.07–1.49)	0.15

SHR, Subdistribution hazard ratio; BMI, body mass index; LLM, Lipid-lowering medications.

*Indicates that the corresponding *P* value is statistically significant (*P* < 0.05).

**Table 7:** Boosting fine-gray model results.

Characteristics	Coef	SHR (95%CI)	*P* Value
Gender (Male)	0.25	1.28 (0.95–1.73)	0.11
Age (Year)	−0.02	0.98 (0.97–0.99)	0.003*
Education level			
6–9 years	−0.24	0.78 (0.54–1.13)	0.19
9–12 years	−0.58	0.56 (0.36–0.88)	0.012*
>12 years	−0.37	0.69 (0.44–1.09)	0.11
Blood pressure	−0.21	0.81 (0.6–1.1)	0.18
Heart rate	−0.29	0.75 (0.5–1.13)	0.17
24 h urine output			
Low	−0.28	0.76 (0.4–1.44)	0.39
Normal	−0.34	0.72 (0.41–1.26)	0.24
High	0.15	1.16 (0.57–2.35)	0.68
BMI	0.05	1.05 (1–1.1)	0.039*
Primary renal disease			
Diabetic kidney disease	−0.08	0.93 (0.44–1.95)	0.84
Others	0.25	1.29 (0.8–2.07)	0.3
Unknown	0.15	1.16 (0.81–1.66)	0.42
Hypertension	−0.14	0.87 (0.65–1.16)	0.34
Diabetes mellitus	−0.38	0.69 (0.36–1.31)	0.26
History of urological Surgery	0.79	2.21 (1.25–3.91)	0.007*
History of kidney transplantation	−0.66	0.52 (0.2–1.35)	0.18
History of hemodialysis	−1.38	0.25 (0.07–0.91)	0.035*
Peritoneal dialysis catheter surgery plan (Open surgery)	−0.4	0.67 (0.38–1.19)	0.17
Antidiabetic medications	0.51	1.66 (0.91–3.01)	0.098
LLM	0.24	1.27 (0.84–1.91)	0.25
Anemia-related parameters	0.37	1.44 (0.94–2.214)	0.096
Antihypertensive medications	0.16	1.17 (0.85–1.62)	0.34
History of hospitalization for peritonitis	0.78	2.18 (1.64–2.9)	0.000*
History of hospitalization for cardiovascular disease	−0.23	0.8 (0.54–1.18)	0.26
Nutrition-related parameters	−1.42	0.24 (0.07–0.85)	0.027*
Lipid-related parameters	−1.23	0.29 (0.06–1.39)	0.12

SHR, Subdistribution hazard ratio; BMI, body mass index; LLM, Lipid-lowering medications.

*Indicates that the corresponding *P* value is statistically significant (*P* < 0.05).

### LASSO-cox model

The LASSO-Cox proportional hazards model was built using a two-stage feature-selection workflow integrating LASSO penalized regression and subsequent stepwise regression, with results summarized in Table 8. Factors associated with HD transition included sex, educational level, (BMI), antidiabetic medication use, history of peritonitis-related hospitalization, and nutrition-related parameters. Higher educational attainment and favourable nutritional status exerted protective effects against PD-to-HD conversion. By comparison, male sex, elevated BMI, antidiabetic medication use, and prior peritonitis-associated hospitalization correlated with elevated instantaneous risk of HD transition and were identified as risk-increasing factors.

**Table 8:** LASSO-cox model results.

Characteristics	coef	HR (95%CI)	*P* Value
Gender	0.39	1.47 (1.10, 1.97)	0.01*
Education level			
6–9 years	−0.53	0.59 (0.41, 0.83)	0.003*
9–12 years	−0.75	0.47 (0.31, 0.72)	<0.001*
>12 years	−0.48	0.62 (0.41, 0.93)	0.02*
Antidiabetic medications	0.44	1.55 (1.07, 2.26)	0.02*
BMI	0.04	1.05 (1.01, 1.09)	0.03*
History of hospitalization for peritonitis	0.58	1.79 (1.37, 2.33)	<0.001*
History of hospitalization for cardiovascular disease	−0.30	0.74 (0.50, 1.10)	0.14
Nutrition-related parameters	−2.10	0.12 (0.04, 0.40)	<0.001*
Lipid-related parameters	−1.06	0.35 (0.08, 1.43)	0.14

HR, Hazard ratio; BMI, body mass index; LLM, Lipid-lowering medications.

*Indicates that the corresponding *P* value is statistically significant (*P* < 0.05).

### Model performance

Time-dependent AUC curves and C-indices of the four models (CSH model, penalized Fine-Gray model, boosting Fine-Gray model and LASSO-Cox model) are shown in Figures 3, 4. The average AUC of the penalized Fine-Gray model in the training set (0.716) was superior to that of the CSH model (0.696) and comparable to that of the boosted Fine-Gray model (0.718), yet lower than that of the LASSO-Cox model (0.762). However, its average AUC in the test set (0.689) outperforms the other three methods.

**Figure 3:**
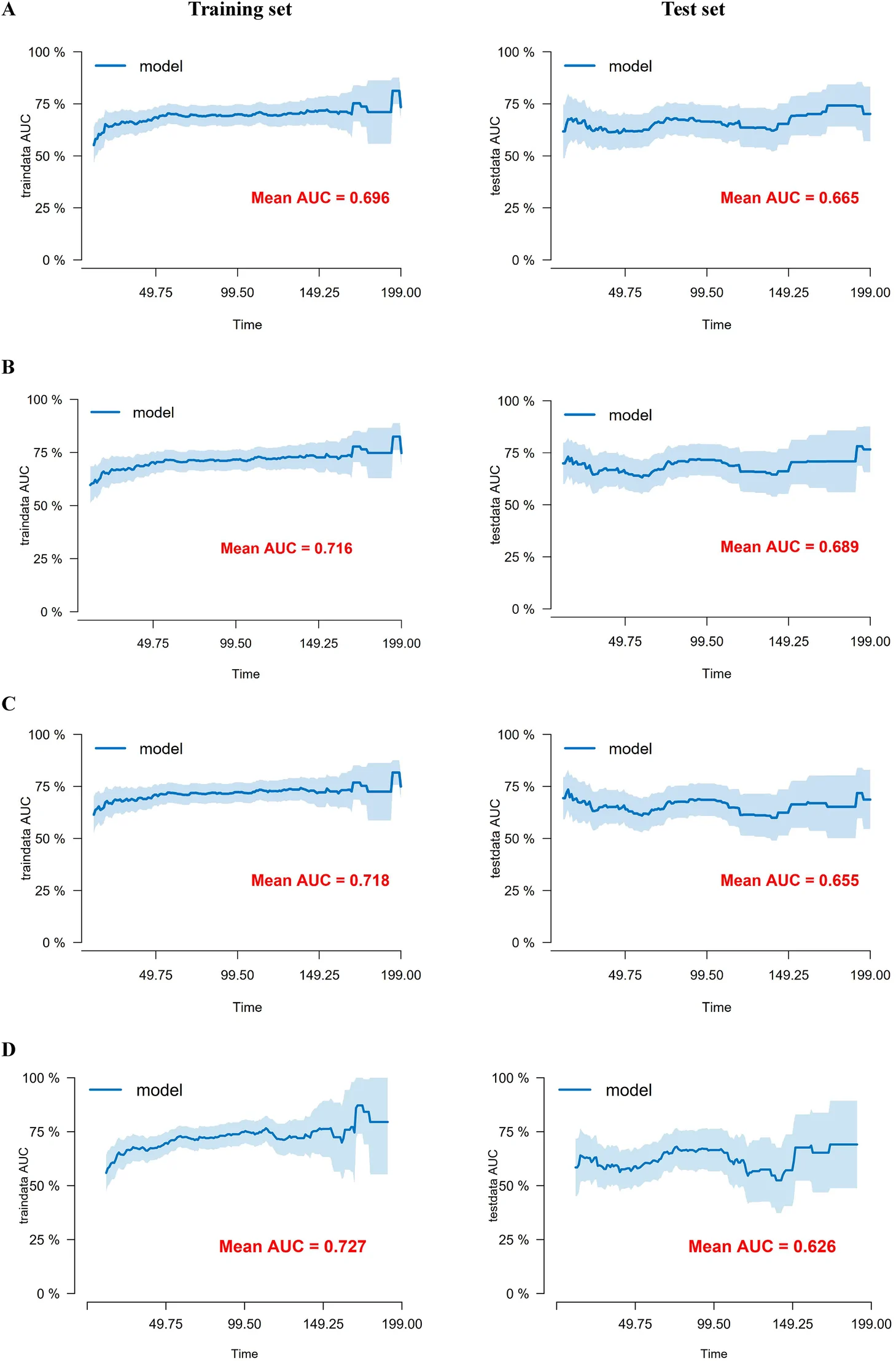
Time-dependent AUC. The left column shows the results of the training set, and the right column shows the results of the test set. The subfigures from top to bottom include the CSH model **(A)**, penalized Fine-Gray model **(B)**, boosting Fine-Gray model **(C)** and LASSO-Cox model **(D)**, respectively. AUC, area under the curve; CSH, cause-specific proportional hazards.

**Figure 4:**
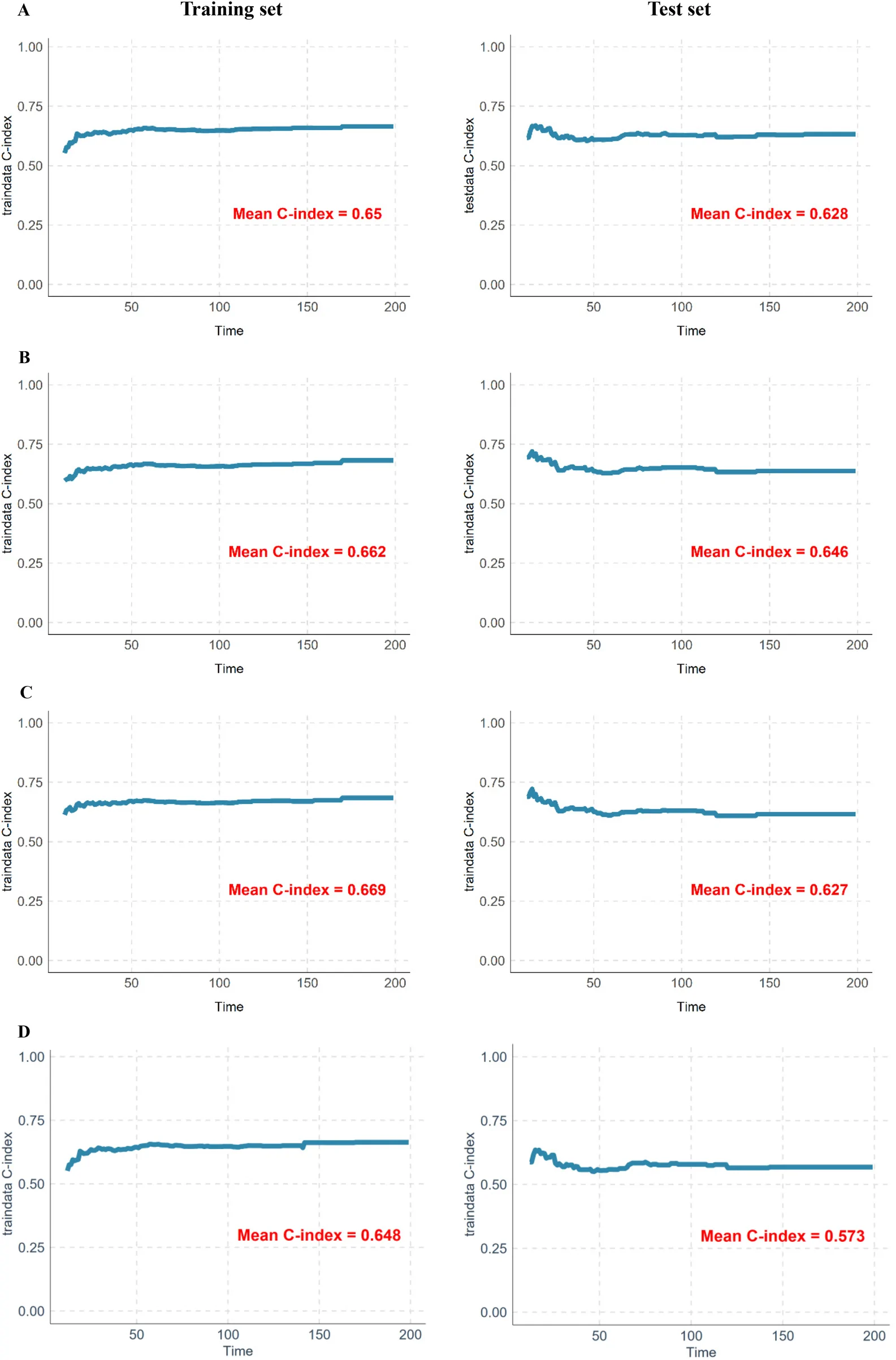
Time-dependent C-index. The left column shows the results of the training set, and the right column shows the results of the test set. The subfigures from top to bottom include the CSH model **(A)**, penalized Fine-Gray model **(B)**, boosting Fine–Gray model **(C)** and LASSO-Cox model **(D)**, respectively. C-index, concordance index; CSH, cause-specific proportional hazards.

Regarding the C-index, the average C-index of the penalized Fine-Gray model in the training set (0.662) was still comparable to that of the other three methods, whereas its average C-index in the test set (0.646) was also higher than that of the CSH model (0.628), the boosting Fine-Gray model (0.627) and the LASSO-Cox model (0.573).

This observation highlights that competing risk models exhibit better generalizability than the conventional LASSO-Cox model in the presence of competing endpoints. Additionally, the number of features incorporated into the penalized Fine-Gray model was lower than that of the boosting Fine-Gray model, as shown in Table 5. As a result, the penalized Fine-Gray model emerged as the best among the four models, demonstrating both favorable predictive performance and clinical applicability.

Proportional subdistribution hazards (PSH) assumption diagnostics were further performed for this selected optimal penalized Fine-Gray model. PSH assumption violations were identified for three covariates: normal 24 h urine output, peritonitis hospitalization, and nutrition-related parameters. Since 24 h urine output was non-significant across all levels in the main model, remedial attempts mainly focused on peritonitis hospitalization and nutrition-related parameters.

Stratified subgroup analyses were explored to resolve assumption violation. Stratification by peritonitis hospitalization failed to satisfy the PSH assumption within subgroups. By contrast, stratification based on a clinically-derived nutrition cut-point (0.5077 in our cohort, corresponding to pre-albumin 300 mg/L and serum albumin 35 g/L) yielded two subgroups in which all covariates fulfilled the PSH assumption. The comparison of mean time-dependent AUC and C-index for the unstratified Penalized Fine-Gray model and nutritional-subgroup Fine-Gray models is shown in Table 9. The mean time-dependent AUC of the model in the training set of the normal nutritional status subgroup (0.763) was superior to that in the test set (0.609). For the malnutrition subgroup, the mean time-dependent AUC in the training set (0.714) was marginally higher than that in the test set (0.691). Regarding the C-index, the mean C-index of the model in the training set of the normal nutritional status subgroup (0.686) exceeded that in the test set (0.579), whereas the mean C-index in the training set of the malnutrition subgroup (0.661) was comparable to that in the test set (0.640). Model evaluation for the two nutritional subgroups revealed unstable generalizability in the well-nourished subgroup with substantial train-test performance divergence. Thus, subgroup-based models were treated only as exploratory alternatives. The main penalized Fine-Gray model, nomogram and variable selection workflow retained the full dataset with nutrition-related parameters as a continuous variable. Full results of PSH testing and subgroup regression outputs are summarized in Supplementary Tables S2, S3.

**Table 9:** Comparison of mean time-dependent AUC and C-index between unstratified penalized fine-gray model and nutritional subgroup fine-gray models.

Metrics	Nutrition status (normal) subgroup		Nutrition status (malnutrition) subgroup		Penalized Fine-Gray model	
	Training set	Test set	Training set	Test set	Training set	Test set
AUC	0.763	0.609	0.714	0.691	0.716	0.689
C-index	0.686	0.579	0.661	0.640	0.662	0.646

AUC, area under the curve.

To further assess the predictive efficacy of the optimal model across distinct time horizons, three representative time points (3, 6, and 9 years) were selected for analysis, as shown in Figure 5. Based on the receiver operating characteristic (ROC) and calibration curves generated at these three time points, the penalized Fine-Gray model demonstrated robust predictive performance across the entire observation period.

**Figure 5:**
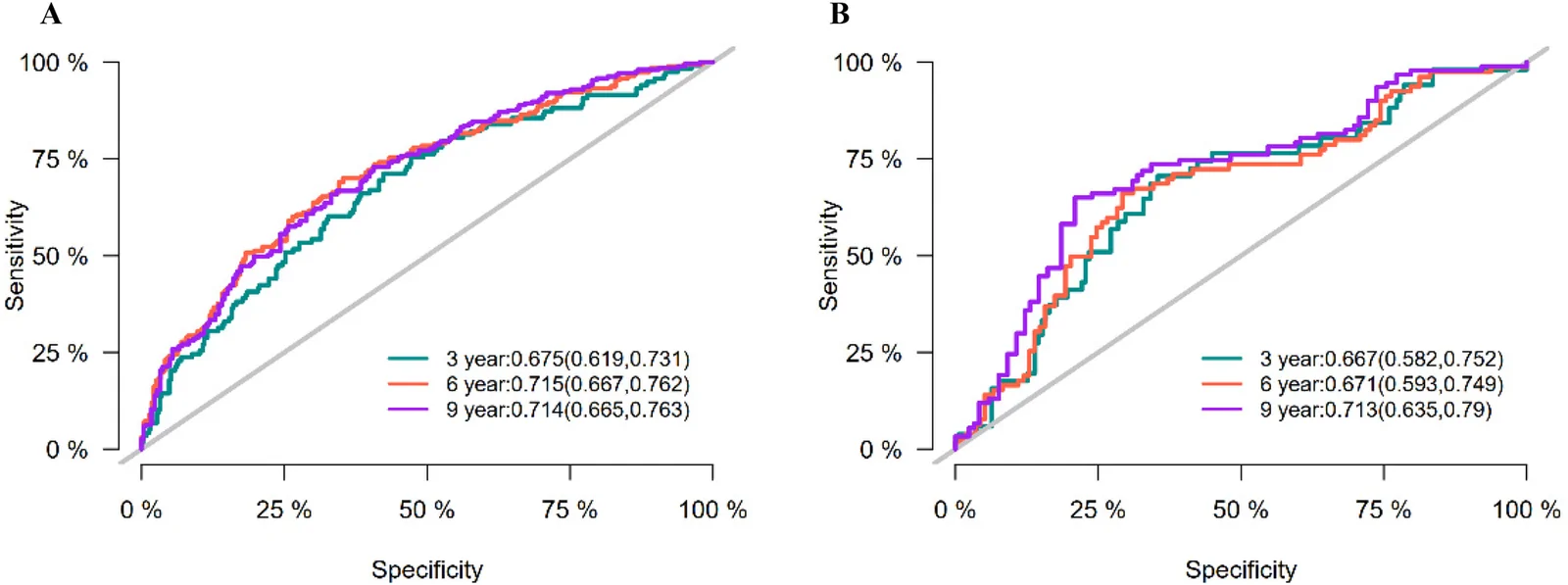
ROC curves for 3, 6, and 9 years. The left panel shows the results of the training set **(A)**, and the right panel shows the results of the test set **(B)**. ROC, receiver operating characteristic.

In terms of the ROC curves, the AUC values for the training cohort at 3, 6, and 9 years were 0.675, 0.715, and 0.714, respectively; the corresponding AUC values for the validation cohort were 0.667, 0.671, and 0.713. The minimal discrepancy in predictive performance between the training and validation sets indicates that the model's capacity to predict event risks at different time points is not only effective in the training cohort, but also possesses satisfactory generalization stability in independent samples.

Regarding the calibration curves (Figure 6), the calibration points of the training cohort at each time point were closely aligned with the diagonal reference line. For the validation cohort, although minor fluctuations were observed at the calibration points, they generally remained proximal to the diagonal. This finding suggests a moderate level of consistency between the model-predicted probabilities and actual occurrence of HD Transition events from PD.

**Figure 6:**
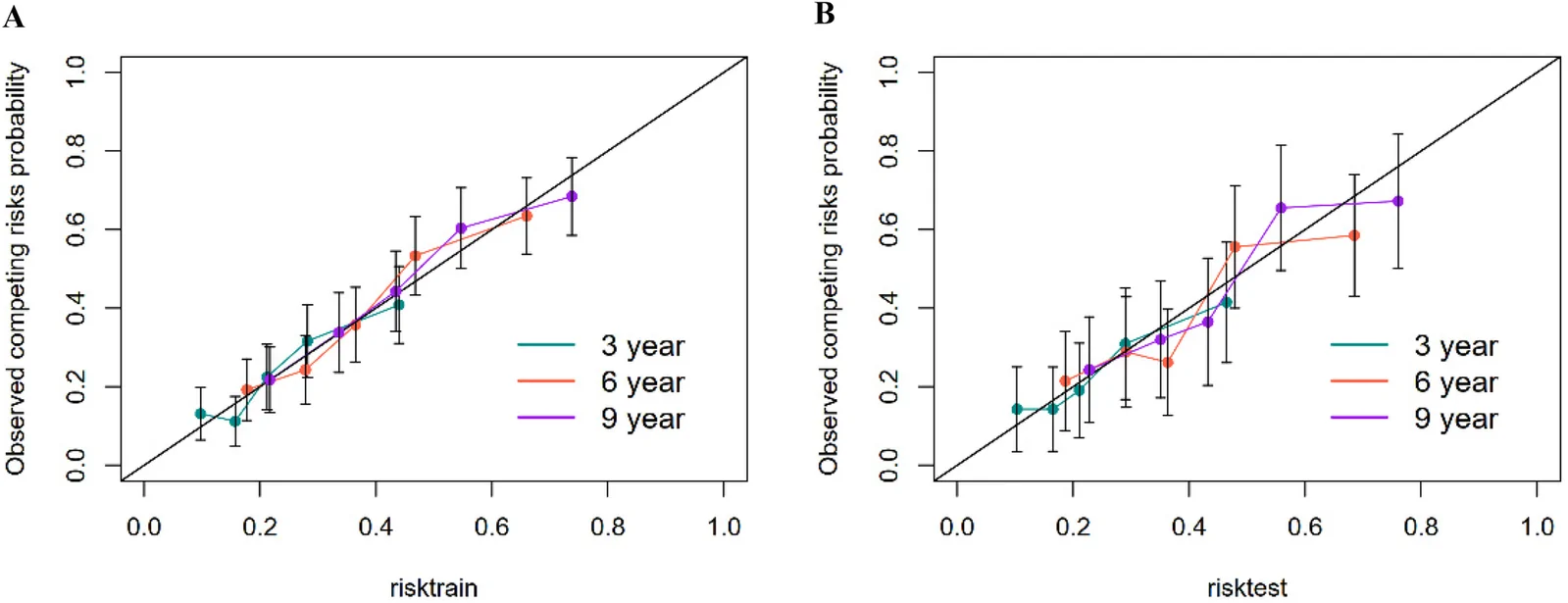
Calibration curves for 3, 6, and 9 years. The left panel shows the results of the training set, **(A)** and the right panel shows the results of the test set **(B)**. The horizontal axis represents the predicted cumulative incidence, and the vertical axis represents the actual cumulative incidence for HD transition events from PD. PD, peritoneal dialysis; HD, hemodialysis.

### Nomogram

We constructed a nomogram to quantify the multivariate risk contributions in the Fine-Gray model into intuitive scores, which can be rapidly mapped to the cumulative incidence of patients with PD progressing to HD at 3, 6, and 9 years (Figure 7). A representative patient case was utilized to illustrate the practical application of the nomogram, with the corresponding clinical characteristics summarized as follows: male, 60 years of age, ≤6 years of education, normal heart rate, reduced 24 h urine output, BMI of 20.4 kg/m^2^, no history of urological surgery or HD, open surgical catheterization for PD access, no LLM, regular use of antihypertensive and anemia medications, and a history of hospitalization for both peritonitis and cardiovascular disease. The nutrition-related parameter and lipid-related parameters were recorded as 0.44 and 0.64, respectively.

**Figure 7:**
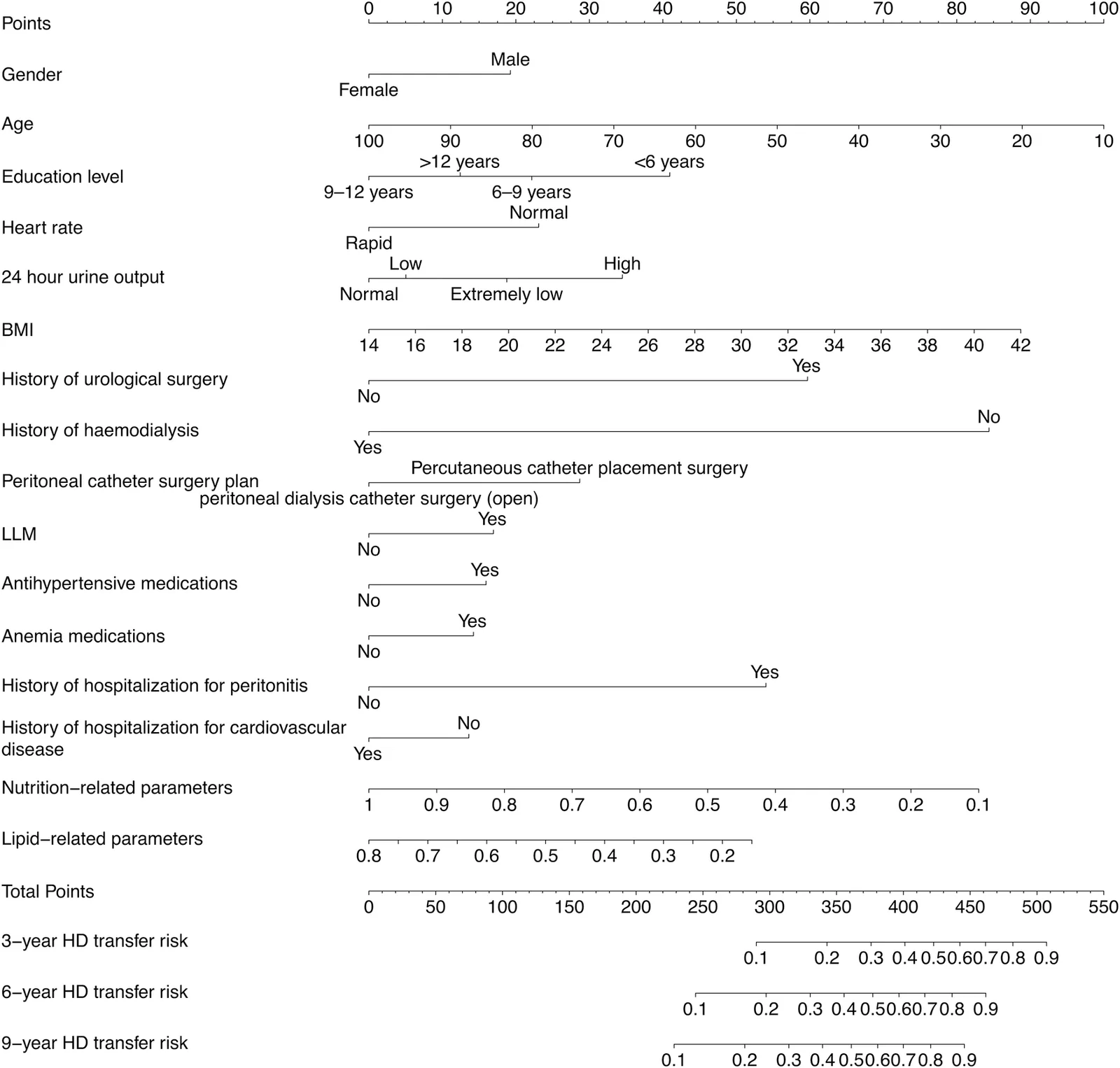
Competing risks nomogram for 3, 6, and 9 years based on the penalized fine–gray model. For each clinical characteristic of a patient, draw a vertical line upward to the top “Points” scale to obtain the corresponding score for that variable. Sum the individual scores of all variables to calculate the “Total Points”. A vertical line drawn downward from the total points to the bottom 3-year, 6-year and 9-year risk scales yields the estimated cumulative probability of PD-to-HD transition at each corresponding time point.

By adding the scores assigned to each of the above characteristics in the nomogram, the patient achieved a total score of approximately 398 (Figure 8). Based on the risk probability calibration of the nomogram, the cumulative risk of transfer from PD to HD was estimated to be 39% at 3 years, rising to 60% at 6 years, and further increasing to 69% at 9 years. These findings demonstrate a progressive elevation in the patient's HD Transition risk over time, with medium- and long-term risk levels classified as moderate to high. This highlights the need for targeted interventions for the relevant risk factors and intensified long-term follow-up monitoring.

**Figure 8:**
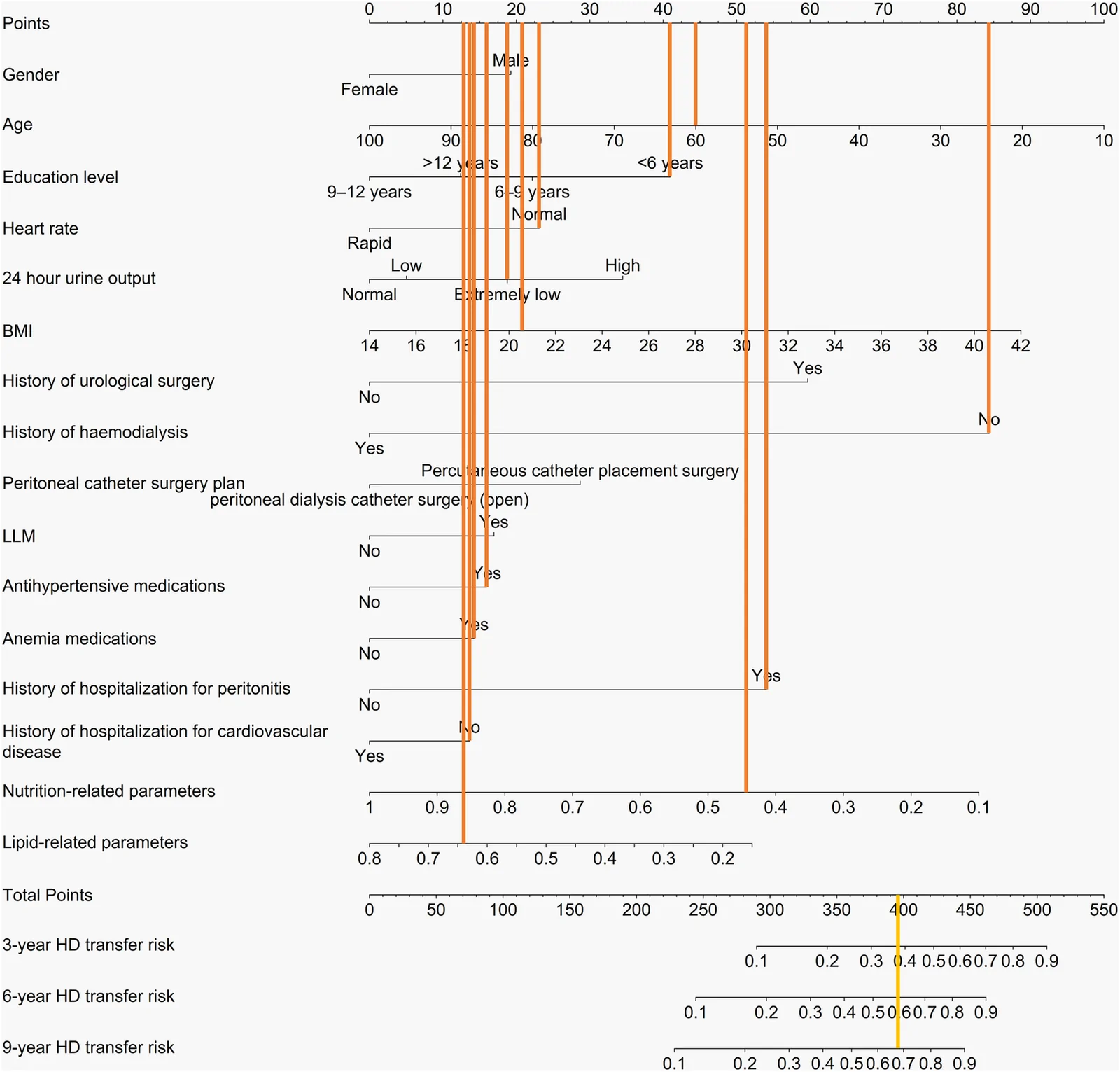
Example of using a line chart. This figure illustrates the full workflow of the nomogram using one representative patient. The clinical characteristics of this illustrative patient are as follows: male, 60 years of age, ≤6 years of education, normal heart rate, reduced 24-h urine output, BMI of 20.4 kg/m^2^, no history of urological surgery or HD, open surgical catheterization for PD access, no LLM, regular use of antihypertensive and anemia medications, and a history of hospitalization for both peritonitis and cardiovascular disease. The nutrition-related parameter and lipid-related parameters were recorded as 0.44 and 0.64, respectively. Each variable of this patient was projected onto the point scale, yielding a total score of approximately 398 points. Projecting downward from this total score, the estimated cumulative risk of HD transition was around 39% at 3 years, 60% at 6 years, and 69% at 9 years. The orange vertical lines in the figure identify the individual point score for each clinical characteristic, and the yellow vertical lines map the total score to the predicted 3-, 6-, and 9-year risks of transitioning to hemodialysis.

Patients were stratified into low risk, middle risk, and high risk subgroups based on tertiles of nomogram total scores from the training cohort. low risk (<327 points), middle risk (327 ≤score <366 points), and high risk (≥366 points). Cumulative incidence function curves of the target event were plotted for each subgroup within the test cohort, and inter-group discriminatory performance was assessed using Gray's test. Significant between-group differences were detected for the cumulative incidence of both the target event (*P* < 0.001) and the competing event (*P* = 0.013). As shown in Supplementary Figure S1, the CIF curves displayed the highest cumulative incidence of the target event in the high risk subgroup. Lower cumulative incidences were observed in the middle risk and low risk subgroups in sequence. A transient minor curve crossing was noted between the low risk and middle risk groups during early follow-up. The curves diverged after 60 months. Risk ordering remained stable thereafter. Adequate risk-stratification capacity of the nomogram was demonstrated.

As shown in Figure 9, stratified by the risk of PD-to-HD transition derived from the nomogram, we have proposed targeted clinical action strategies for patients at different risk levels. for example, low-risk patients may continue standard PD care with modification of modifiable factors and routine patient education every 3–6 months, whereas high-risk patients may require multidisciplinary evaluation, early HD access planning, and monthly or more frequent monitoring. This risk stratification can provide valuable insights for the daily operational management of dialysis wards.

**Figure 9:**
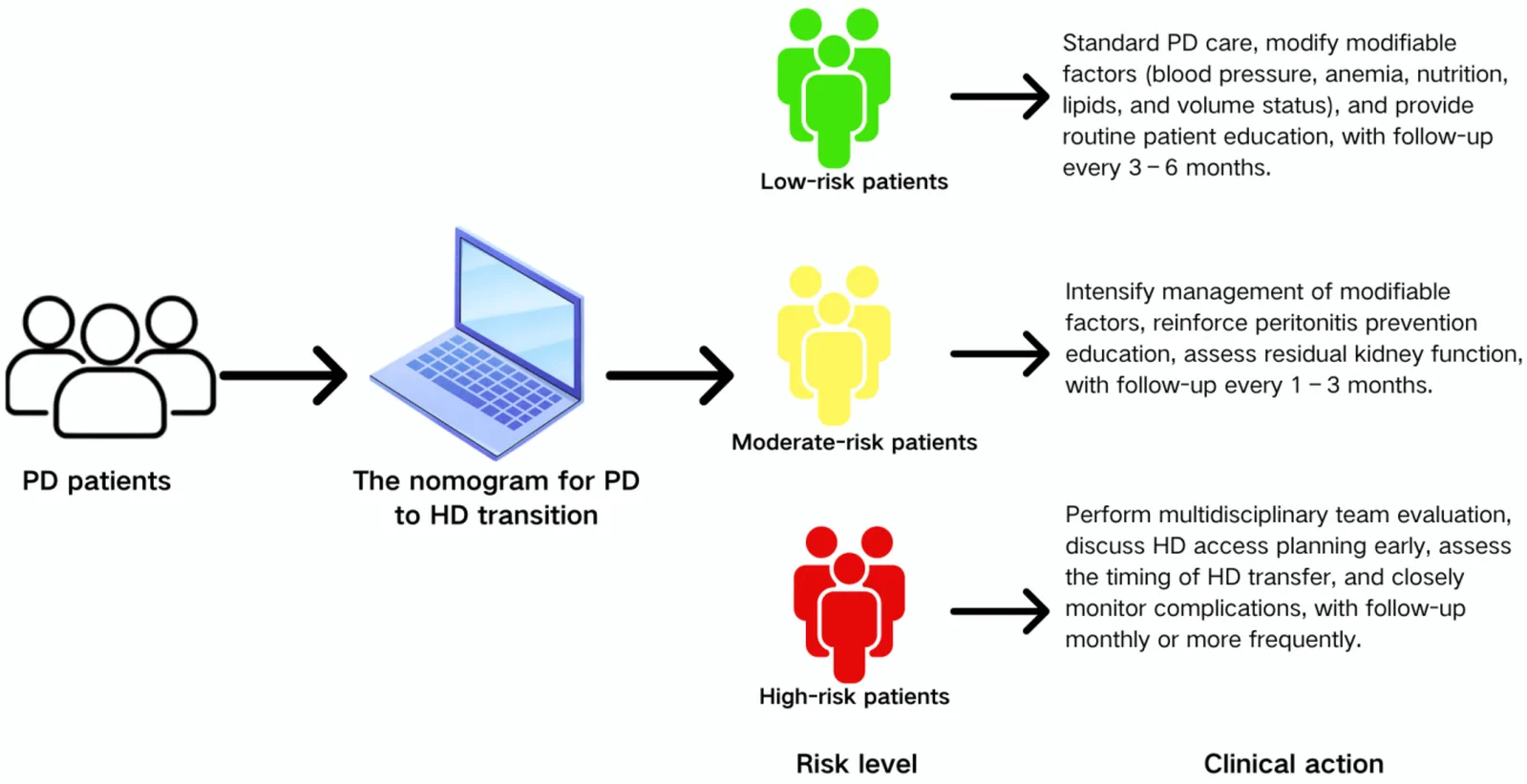
Clinical action algorithm based on the nomogram for PD to HD transition. The nomogram integrates key clinical and laboratory parameters to stratify PD patients by risk of HD transfer. Based on individualized risk estimation, patients are classified into low-, intermediate-, and high-risk groups, and management strategies and follow-up schedules are tailored accordingly. PD, peritoneal dialysis; HD, hemodialysis. This algorithm is intended to support, rather than replace, individualized clinical decision-making, and should be integrated with patient preferences, center resources, and dynamic clinical changes.

## Discussion

### Our findings

The study data are characterized by three distinct outcomes. Traditional Cox proportional hazards models focus exclusively on the transition from PD to HD and treat death as a censored event, which consequently leads to an overestimation of patients' HD transition risk. In the outcomes of patients with PD, the transition to HD, death, and remaining event-free until the end of the follow-up constitute mutually exclusive competing risks. When such competing events occur, the application of competing risk models for analysis is more appropriate. The most commonly used competing risk models include the cause-specific proportional hazards (CSH) model and the Fine-Gray model, both of which address the limitations of the Cox model. In this study, we simultaneously applied the CSH and Fine-Gray models for multivariate analysis to identify factors influencing the cumulative incidence of HD transition in PD patients. In addition, a conventional LASSO-Cox model reproduced from existing literature was incorporated for comparison (26). Model construction was optimized using a variety of feature selection methods, including LASSO, SCAD, MCP-penalized regression, and boosting algorithms. By comparing the time-dependent AUC and C-index, the penalized Fine-Gray model was identified as the optimal predictive tool, and a clinically useful nomogram was subsequently developed.

### Potential mechanisms of PD to HD transition

All models classified BMI, history of hospitalization for peritonitis, and nutrition-related parameters as significant predictors, consistent with the findings of previous studies (27, 28). A history of hospitalization for peritonitis was a severe complication of PD. It can directly render PD treatment unsustainable, requiring the removal of the PD catheter and conversion to temporary HD support. Alternatively, it may cause long-term damage to the structure and function of the peritoneum, gradually impairing dialysis efficacy, and ultimately leading to failure of the PD technique, which in turn necessitates permanent conversion to HD therapy (29). Patients with PD and a higher BMI are at greater risk of PD-associated peritonitis, residual renal function loss, and increased hospitalization frequency (30), all of which are associated with the discontinuation of PD. Poor nutrition can impair immune function, weaken tissue repair ability, and reduce resistance and recovery ability to infection; malnutrition is also a significant risk factor for peritonitis in patients with PD, while recurrent peritonitis is a risk factor for PD withdrawal (31–33).

In addition, the penalized Fine-Gray model identified several other significant predictors, including age, education level, history of urological surgery, and history of HD. Age was a significant factor influencing the progression from PD to HD. Younger adults often face greater social pressures such as employment, psychological issues, self-image, and medication adherence. The interplay of these factors could lead to earlier or more passive withdrawal from peritoneal dialysis in favor of hemodialysis. A mixed-methods study of 470 patients with PD showed that young adults (aged 15–30 years) were at a higher risk of transitioning to HD, mainly due to infections, noncompliance, and mechanical issues (34). This study found that a lower education level is a significant risk factor for peritonitis and technical failure (35). The underlying reason for this may be that lower educational levels are often associated with poorer cognition, lower income, and poorer treatment adherence (36, 37). We also identified a history of urological surgery as a risk factor for progression from PD to HD. Patients with urological diseases such as urinary tract tumors, urethral malformations, or urethral obstruction often exhibit poor general conditions and multiple comorbidities (38, 39), constituting a high-risk population for PD discontinuation. Therefore, these risk factors should be considered when initiating PD. We also observed that patients with a history of HD had a lower risk of transitioning from PD to HD. This apparent protective association is likely not a direct protective effect of prior HD itself, but rather a reflection of selection bias: patients with a history of HD may be more familiar with dialysis procedures and the healthcare system and may have better adherence, more regular follow-up, and better self-management, thereby reducing the risk of PD technique failure. It is well known that HD patients require to 2–3 sessions of central dialysis per week, which significantly affects their daily lives and work. Therefore, for PD patients with a history of HD, a PD regimen with higher social regression rates and a lower economic burden is preferred (40).

### Study strengths and limitations

This study adopted a competing risk analysis, which is more suitable for the outcome characteristics (i.e., coexistence of HD transition, death, and no events). To alleviate the collinearity of the clinical variables, composite domain scores were calculated using the CRITIC weighting method. The optimal penalized Fine-Gray model was identified through comprehensive model optimization and validation, and was characterized by a concise set of predictors that effectively reduced the risk of overfitting. Additionally, the nomogram derived from the optimal model enables clinicians to quickly quantify the 3-, 6-, and 9-year cumulative risks of HD transition for individual patients on PD and provides corresponding clinical recommendations based on risk stratification, providing a reference tool for personalized risk stratification management.

This study has several limitations. First, as a single-center retrospective study, the sample source was subject to inherent limitations. The external generalizability of the results requires further verification using multi-center, large-sample data. Second, there were potential deficiencies at the data level; the missing rate of some clinical indicators exceeded 30%. Although multiple linear regression imputation and k-nearest neighbor imputation were used for data processing, imputation bias may still exist, which could weaken the accurate characterization of the association intensity between the predictors and the target event. Third, the study did not incorporate dynamic changes in clinical indicators during follow-up, which may limit the comprehensiveness of the risk assessment. Fourth, partial violation of the PSH assumption was observed for several covariates in the final penalized Fine-Gray model. This issue may exert a moderate influence on model predictive accuracy. Advanced competing-risk methodologies that account for time-varying covariate effects deserve consideration in future investigations to further improve predictive performance.

## Conclusions and clinical implications

The penalized Fine-Gray model was ultimately identified as the optimal predictive tool for PD-to-HD transition, and the nomogram we constructed based on this model exhibits favorable clinical applicability. Future studies should focus on multi-center prospective validation and incorporate dynamic follow-up indicators to further enhance the clinical utility of the predictive models.

## Data Availability

The original contributions presented in the study are included in the article/Supplementary Material, further inquiries can be directed to the corresponding author/s.
