# Supplementary material for: Optimal prediction of peritoneal dialysis to hemodialysis transition: a penalized fine-gray model
Source: Front Med (Lausanne). 2026 Oct 21;13:1843381. doi: 10.3389/fmed.2026.1843381 (PMC13730853; doi:10.3389/fmed.2026.1843381)
Supplement: Supplementary file 1 [file Supplementaryfile1.docx]

**Supplementary Table 1** Summary of missing data and imputation methods

| Characteristics | Missing rate (%) | Imputation method |
| --- | --- | --- |
| Serum sodium | 0.14 | Mean imputation |
| Serum potassium | 0.14 |  |
| Serum urea nitrogen | 0.43 |  |
| Serum albumin | 0.43 |  |
| Serum chloremia | 0.57 |  |
| Carbon dioxide combining power | 0.57 |  |
|  |  |  |
| Calcium-phosphorus product | 6.43 | K-nearest neighbor（k=5） |
| Transferrin saturation | 10.29 |  |
| Intact parathormone | 10.86 |  |
| Prealbumin | 18.14 |  |
| Ferritin | 18.86 |  |
| Alkaline phosphatase | 20.14 |  |
|  |  |  |
| Total cholesterol | 10.71 | Multiple linear regression imputation |
| Triglyceride | 10.86 |  |
| Low-density lipoprotein | 10.86 |  |
| High-density lipoprotein | 11.29 |  |
| Fasting glycemic control | 20.29 |  |
| Myoglobin | 37.00 |  |
| Troponin | 37.00 |  |
| Creatine kinase | 37.14 |  |
| Glycosylated hemoglobin | 55.57 |  |

**Supplementary Table 2** Results of proportional subdistribution hazards assumption tests

| Characteristics | Penalized Fine-Gray model | History of hospitalization for peritonitis (Yes) subgroup | History of hospitalization for peritonitis (No) subgroup | Nutrition status (normal) subgroup | Nutrition status (malnutrition) subgroup |
| --- | --- | --- | --- | --- | --- |
| Gender | 0.391 | 0.080 | 0.753 | 0.354 | 0.701 |
| Age | 0.241 | 0.438 | 0.556 | 0.719 | 0.560 |
| Education level |  |  |  |  |  |
| 6-9 years | 0.532 | 0.536 | 0.196 | 0.749 | 0.207 |
| 9-12 years | 0.196 | 0.160 | 0.452 | 0.373 | 0.495 |
| ＞12 years | 0.959 | 0.793 | 0.554 | 0.746 | 0.534 |
| Heart rate | 0.757 | 0.826 | 0.280 | 0.335 | 0.392 |
| 24-Hour urine output |  |  |  |  |  |
| Low | 0.020 | 0.390 | 0.168 | 0.105 | 0.150 |
| Normal | 0.387 | 0.763 | 0.655 | 0.462 | 0.609 |
| High | 0.627 | 0.491 | 0.465 | 0.901 | 0.412 |
| BMI | 0.282 | 0.087 | 0.285 | 0.580 | 0.358 |
| History of urological surgery | 0.227 | 0.211 | 0.373 | 0.315 | 0.338 |
| History of hemodialysis | 0.223 | 0.307 | 0.266 | 0.431 | 0.269 |
| Peritoneal dialysis catheter surgery plan(Open surgery) | 0.179 | 0.090 | 0.367 | 0.100 | 0.332 |
| LLM | 0.439 | 0.250 | 0.571 | 0.869 | 0.518 |
| Antihypertensive Medications | 0.640 | 0.801 | 0.825 | 0.792 | 0.835 |
| Anemia-related parameters | 0.603 | 0.471 | 0.809 | 0.693 | 0.900 |
| History of hospitalization for peritonitis | 0.033 | - | - | 0.097 | 0.293 |
| History of hospitalization for cardiovascular disease | 0.415 | 0.622 | 0.615 | 0.392 | 0.644 |
| Nutrition-related parameters | 0.017 | 0.023 | 0.155 | - | - |
| Lipid-related parameters | 0.702 | 0.166 | 0.518 | 0.278 | 0.370 |
| Global test | 0.046 | 0.336 | 0.502 | 0.518 | 0.414 |

BMI, body mass index; LLM, Lipid-lowering medications.

**Supplementary Table 3** Fine-Gray model results for the subgroup of patients with normal nutrition status

|  | Nutrition status (normal) subgroup | | Nutrition status (malnutrition) subgroup | |
| --- | --- | --- | --- | --- |
| Characteristics | coef | sHR (95%CI) | coef | sHR (95%CI) |
| Gender | 0.66* | 1.93 (1.17-3.19) | 0.03 | 1.04 (0.70-1.53) |
| Age | -0.01 | 0.99 (0.97-1.01) | -0.02* | 0.99 (0.97-1.00) |
| Education level |  |  |  |  |
| 6-9 years | -0.61 | 0.54 (0.29-1.01) | -0.14 | 0.87 (0.55-1.37) |
| 9-12 years | -1.27* | 0.28 (0.14-0.58) | -0.27 | 0.77 (0.44-1.35) |
| ＞12 years | -0.90* | 0.41 (0.19-0.88) | -0.21 | 0.81 (0.46-1.44) |
| Heart rate | -0.90* | 0.41 (0.19-0.85) | -0.02 | 0.98 (0.59-1.64) |
| 24-Hour urine output |  |  |  |  |
| Low | -0.91 | 0.40 (0.12-1.36) | -0.04 | 0.96 (0.47-1.95) |
| Normal | -0.73 | 0.48 (0.18-1.32) | -0.34 | 0.71 (0.37-1.36) |
| High | -0.56 | 0.57 (0.17-1.87) | 0.38 | 1.46 (0.63-3.41) |
| BMI | 0.03 | 1.03 (0.96-1.11) | 0.05 | 1.06 (1.00-1.12) |
| History of urological surgery | 1.05 | 2.85 (0.70-11.61) | 0.76* | 2.14 (1.13-4.08) |
| History of hemodialysis | -1.23 | 0.29 (0.06-1.54) | -1.26 | 0.28 (0.07-1.23) |
| Peritoneal dialysis catheter surgery plan(Open surgery) | -0.29 | 0.75 (0.29-1.91) | -0.65 | 0.52 (0.25-1.07) |
| LLM | 0.48 | 1.61 (0.75-3.45) | 0.24 | 1.27 (0.76-2.13) |
| Antihypertensive Medications | -0.13 | 0.88 (0.43-1.78) | 0.46 | 1.59 (0.94-2.71) |
| Anemia-related parameters | 0.33 | 1.40 (0.77-2.52) | 0.08 | 1.09 (0.73-1.63) |
| History of hospitalization for peritonitis | 0.66* | 1.93 (1.19-3.12) | 0.80* | 2.22 (1.56-3.17) |
| History of hospitalization for cardiovascular disease | -0.12 | 0.89 (0.47-1.66) | -0.24 | 0.79 (0.48-1.29) |
| Nutrition-related parameters | -1.64 | 0.19 (0.01-3.27) | -1.01 | 0.36 (0.06-2.37) |

SHR, Subdistribution hazard ratio; BMI, body mass index; LLM, Lipid-lowering medications.
