# Supplementary material for: Optimal prediction of peritoneal dialysis to hemodialysis transition: a penalized fine-gray model
Source: Front Med (Lausanne). 2026 Oct 21;13:1843381. doi: 10.3389/fmed.2026.1843381 (PMC13730853; doi:10.3389/fmed.2026.1843381)
Supplement: Supplementary file 2 [file Supplementaryfile2.docx]

**Supplementary Figure 1** Cumulative incidence curves for the target event in low-risk, middle-risk and high-risk subgroups
